# [Expression of Concern] siRNA targeting of Trop2 suppresses the proliferation and invasion of lung adenocarcinoma H460 cells

**DOI:** 10.3892/etm.2025.12967

**Published:** 2025-09-09

**Authors:** Xiao-Yan Gao, Ye-Han Zhu, Li-Xin Zhang, Hui-Yu Lu, Ai-Gui Jiang

Exp Ther Med 10:429–434, 2015; DOI: 10.3892/etm.2015.2530

Following the publication of this paper, it was drawn to the Editor's attention by a concerned reader that, for the Transwell migration assay experiments shown in Fig. 5, the ‘Ctrl’ (Fig. 5A) and ‘rAd5-Ctrl-siRNA’ (Fig. 5B) data panels appeared to be overlapping, including a clockwise rotation of the panel through 180° comparing Fig. 5A with Fig. 5B, suggesting that data which were intended to show the results from differently performed experiments had apparently been derived from the same original source. A subsequent analysis of the data in this paper made by the Editorial Office revealed that the cell proliferation assay data in Fig. 4Aa and 4Ba contained an overlapping section, albeit the images were rotated through 90° in one of the data panels relative to the other. The authors were contacted by the Editorial Office to offer an explanation for these apparent anomalies in the presentation of the data in this paper, although up to this time, no response from them has been forthcoming. Owing to the fact that the Editorial Office has been made aware of potential issues surrounding the scientific integrity of this paper, we are issuing an Expression of Concern to notify readers of this potential problem while the Editorial Office continues to investigate this matter further.

